# Tuberculosis of the Intestine

**Published:** 1923-06

**Authors:** D. A. Stewart


### TUBERCULOSIS OF THE INTESTINE—THE ULCERATIVE FORM AS A PHASE OF PULMONARY TUBERCULOSIS

---

BY D. A. STEWART

---

In the past five years a series of masterly papers and addresses by Archibald, Pirie, Brown and Sampson, and Carman, with a considerable literature besides, has brought this phase of tuberculosis, clearly, almost dramatically, before both physicians and surgeons, I venture to bring the subject again before you to discuss the question of how close the association is between the disease manifesting itself in the lung and in the intestine.

Looking backward over what experience we each may have had in the treatment of tuberculosis patients; considering the frequency, the early appearance, and the persistence of intestinal symptoms; considering the old dicta that the road to recovery lies through the stomach; that the best friend of the consumptive is a good digestion; that poor eaters do badly; that digestion is the keystone of the prognostic arch; that a flux of the bowels tends to a fatal outcome, should we not be on the outlook for early and definite complications in the intestinal tract? Among eighty-nine unselected cases of pulmonary tuberculosis, many of them early, Lawrason Brown found four with definite enteritis, roughly four per cent., and two suspected, roughly two per cent. In the Manitoba Sanatorium we deal with patients of farther advanced types than Brown's. Of five hundred and sixty who were definitely tuberculous in the period under consideration —a little over two years—only six per cent. were classed, as to their pulmonary disease, as incipient; sixteen per cent. as moderately advanced, and seventy-eight per cent. as far advanced. Of the far advanced, one-third, that is twenty-three per cent. of all admitted, were sub-classed as "apparently hopeless."

All five hundred and sixty patients were questioned as to intestinal symptoms, and fully examined; a barium investigation was made on suspicion only. Positive symptoms and also positive barium signs were required for a definite diagnosis. In this body of patients, then, out of five hundred and sixty, two hundred and nine, or about thirty-seven per cent., were definitely suspected of intestinal disease. Of these, sixty-three, or thirty per cent., were considered fairly negative, forty-three, or twenty per cent., doubtful or not proven, and one hundred and three, or fifty per cent. of those suspected of intestinal, were regarded as positive. Of the five hundred and fifty, then, thirty-seven per cent. were suspected of intestinal tuberculosis, and eighteen per cent. diagnosed as having definite intestinal tuberculosis.

Our impression is that in this series we have been altogether too conservative; that we have demanded too much evidence, especially too definite barium signs, before making a diagnosis; that the incident of intestinal disease is really higher in cases of this type than our percentages show; that most of our suspected cases should be considered positive, and that full investigation of the intestinal tract in patients having pulmonary tuberculosis should be a routine. A series of post-mortem examinations by several observers have shown definite tuberculous intestinal ulceration in from seventy-five to ninety per cent. of the bodies of those who have died of pulmonary tuberculosis. Such a high percentage as this at the time of death suggests that enteritis may have given the "knock-out blow."

In the ordinary run of post-mortem material, healed intestinal lesions are found, even in the most bodies of children. Heller and Wagner found twenty-eight foci, evidently primary, and healed, in six hundred autopsies. Opie states that in twenty-five per cent. of all autopsies on British soldiers nodes showing healed ulcers of the bowel were found. Surgeons also find such lesions, healed and unhealed, where there had been no complaint of symptoms. Many such foci must surely be primary. We have then, clinical and pathological evidence of a frequent and close, late and even early association of intestinal with pulmonary tuberculosis. It may be that the pulmonary lesions have too much dominated our picture. Intestinal ulceration has been in the past regarded as a complication only, scarcely looked for or expected until it thrust itself upon our attention. Perhaps we should raise the complication from a subordinate place to an equal or almost equal partnership in crime. Why must it be always pulmonary tuberculosis complicated by intestinal tuberculosis? May it not sometimes be rather intestinal tuberculosis complicated by disease in the lungs, "intestinal and pulmonary" rather than "pulmonary and intestinal," the intestinal disease the earlier of the two, the senior partner? We know it is very often in the late stages the dominant partner. Perhaps the disease entered through the intestine and passed to the lungs and did not enter at the lungs and pass to the intestine.

*Diagnosis*—What of the diagnosis? An ounce of early diagnosis is worth many pounds of attempted cure. A diagnosis well made is treatment well begun. We all know that the old text book symptoms of disease in the intestinal tract, anorexia, even nausea, tenderness and pain, diarrhoea and emaciation. To demand these for the diagnosis of intestinal tuberculosis is like demanding for the diagnosis of pulmonary tuberculosis bubbling and cavernous rales, night sweats and a hectic flush. They are late and prognostic, not early and diagnostic signs. The diagnosis should be made, must be made, long before these appear. Lawrason Brown mentions as early symptoms extreme nervousness, constipation, slight dyspepsia, a feeling of discomfort after meals, gas, failure to gain weight under suitable conditions; a general "not doing well" for which no other special reason can be found. He considers neither pain nor diarrhoea to be really early symptoms. Archibald thinks that any derangement of digestion or of appetite, especially in one who has pulmonary disease, and any pain should always be looked on as possibly due to tuberculosis of the bowels. I am inclined to consider impaired appetite in one who has pulmonary tuberculosis as the most suggestive early symptom. Perhaps one should include any abnormality of appetite. In one of our patients, the usual symptoms of acute intestinal disease found at operation to be very widespread, were preceded by months of ravenous appetite. The nervous organization seems much more profoundly affected by intestinal lesions than by pulmonary, and mental depression is common, although seldom met with in pulmonary disease. Archibald considers typical pains as conclusive in the diagnosis. "When the patient complains of a pain felt in the mid or lower abdomen, coming on at regular intervals during the day, but chiefly from the late forenoon or afternoon on transient often, crampy or stabbing, suggesting gas pains, aggravated by food and relieved by fasting, felt only during part of the day, but persisting from day to day, then one must be very suspicious of tuberculosis. When, in addition, he complains of loss of appetite, of real distaste for food; when he has nausea at times; when he gives up one article of food after another; when he develops a slight fever which is not attributable to his lung condition, and if this persists over three or four weeks, then one may be almost sure of the diagnosis." The later symptoms, which are unfortunately too well known to need mention, may vary according to the localization of the ulcers and other elements and phases.

Any part of the bowel, from the stomach to the anus, may be affected, the order of frequency being apparently the ileocecal region, the ascending colon, jejunum, descending colon, sigmoid and rectum. The ileocecal valve and the caecum would seem to be the storm center. There, Carman says, the ulcers are confluent. Constipation seems characteristic of lesions of the small and diarrhoea of those of the large bowel.

*X-Ray in Diagnosis*—But if the diagnosis is to be made early, before any but vague and slight symptoms have appeared, indeed before definite complaint has been made, the opaque meal and enema must be given at least an equal share with clinical investigation. In our series, the meal was given as a routine, the enema as a secondary procedure, chiefly when in doubt. I am not sure that this order would be approved by all workers. The diseased bowel has patches of ulcerated inner surface; its walls are infiltrated and hypertrophied; and there are constrictions and adhesions which narrow its lumen and make kinks in its course. Naturally, marked abnormalities in the progress of the meal, and in the filling of the bowel by meal and enema, result. Spasm may cause delay, as is, perhaps, a frequent condition when the disease is in the upper bowel; local or general hypermotility may supervene, especially when the disease is in the lower bowel. Every type of pathology in the bowel makes for defective filling. The meal or enema does not show the full expansion, the well-rounded haustrations or the smooth outlines of the normal bowel. But there's the rub. What is the normal bowel? What is a normal nose, or chin, or face, or figure, and what variations in filling can still be considered a normal bowel outline. How few and fine barium variations should be classed as fiagnostic, or how slight symptoms, or how small a total of sign and symptoms? Those who, like Carman, see unselected cases, allow great diagnostic weight, in intestinal conditions, to the presence of pulmonary tuberculosis. We, who are selected series all having pulmonary lesions, perhaps do not allow enough weight in diagnosis for tuberculosis already present in the lungs. Barium is of great help in outlining the large bowel, of very little value in diagnosis of small bowel lesions. If tuberculous ulceration of the bowel has recognizable early stages, if by clinical observation and study, by opaque meals and enemata, and perhaps by other methods yet to be devised we can learn to diagnose at an early stage, and institute treatment at any early stage, we should expect to better the prognosis not only of this one phase of the disease but of the whole general disease—tuberculosis—as well. Late treatment can aim to cure.

Treatment of tuberculosis ulceration of the bowel, which has in the past been chiefly for the relief of symptoms, has included general good conditions, diet, drugs and enemata. To these may now be added light theraph, including perhaps, the X-rays and surgery. The general good conditions, rest, regulation of energy expenditure, fresh air and the various other phases of the routine of a tuberculous patient, become doubly insistent, when the least or earliest sign or even suspicion of intestinal disease arises. Dietetic considerations are rather negative than positive, the choosing of food not irritating or gas producing.

Drugs of almost every kind have been used, antiseptics, sedatives, astringents, opiates, and even lubricants, for the relief of symptoms rather than for the cure of the disease.

*Light Therapy*—The oldest of therapeutic agents has become the most modern also. There is something not new under the sun, light. Artificial light can be applied easier than the sun's rays, and with less risk, more regularly and to patients more ill. The Alpine Lamp seems to give results in intestinal tuberculosis. There is no doubt as to the enthusiastic belief of the patients in its efficacy—and even that is of value.

Our routine, after gradual habituation to the lamp, is forty-eight minutes exposure, twenty-four back and twenty-four front, the lamp at twenty-four inches, every other day, in alternative periods of three weeks. Brown pushes the lamp beyond this limit and exposes to X-rays as well. Of the forty-three doubtful cases in our series, fourteen were unsuitable for lamp treatment on account of progressive lung lesions, and eight for other reasons. Of the twenty-one who had lamp treatment, in sixteen the intestinal symptoms were definitely improved and in five not improved.

Of the one hundred and three positive cases, thirty-nine were hopelessly ill—too ill to be subjected to lamp treatment—and eight have been on this treatment only a short time. Of the fifty-six positive cases and twenty-one doubtful cases, seventy-seven in all, who had lamp treatment long enough to give some idea of results an analysis may be made. Of seventeen with gross active lung disease and acute active intestinal disease, one improved temporarily, two more permanently (one operated upon also), fourteen did not improve. Of the ten with gross active pulmonary tuberculosis and less active intestinal disease two improved temporarily, eight did not improve. Of three who had pulmonary disease less gross and active, but whose intestinal disease was active, all improved, two after operation. Of the remaining forty-seven whose pulmonary disease was not so gross or active and intestinal disease not more than moderately active, all but five made what is apparently permanent improvement, or recovery, and one was improved temporarily; only four made no improvement.

Of the whole seventy-seven, treated by lamp for a considerable time, many of them with gross pulmonary and intestinal lesions, two-thirds have shown improvement. On the other hand, results of operation are, to say the least, not altogether encouraging, even considering the newness of this method, and that it will likely improve, and that lamp results are so recent as to be scarcely comparable. With these considerations in view Brown reports sixteen cases operated upon of whom three are living and thirteen dead; thirty treated by lamp, of whom, so far, twenty-five are living and five dead; and twenty-nine who had no definite treatment beyond general routine of whom ten are living and nineteen dead. Of seven patients we have sent for operation one favorable and one very unfavorable and indeed unsuitable died within a few days of operation. Two had unilateral exclusion for extensive disease, and are apparently recovering or recovered. Two had disease so widespread above and below the valve that operation was decided against. A third with similar findings is making a good recovery after removal of a troublesome looking appendix.

*Surgery*—Surgical treatment of some forms of intestinal tuberculosis is not new. Hypertrophic conditions, stricture of the small bowel due to ulceration and tuberculous disease in the appendix have long been submitted to operation, but resection of exclusion of an ulcer-studded segment of a tuberculous bowel in order, by putting it at rest, to promote healing, is a comparatively new application of surgery. The choice of operation is influenced less, perhaps, by the state of the bowel than by the condition of the patient. Archibald considers the more complete operations preferable, the choice being excision, then exclusion, bilateral or unilateral. When these are not possible, opening the large bowel and forming an artificial anus, or even the removal of a troublesome appendix, may be palliative, and sometimes of more permanent benefit. Widespread active or progressive lung disease is a contra-indication to operation. Archibald considers that the outcome varies directly with the condition of the lung lesion. Wide involvement of the small bowel, which unfortunately can not be determined at all definitely before operation is also a contra-indication.

Among the various kinds of lesions Archibald considers hypertrophy and stricture to be evidence of general resistance of the body to tuberculosis, and ulceration to be associated with lack of resistance. Operation would, of course, be much more likely to be successful in the former than in the latter type of case. Long duration of long symptoms, especially of discomfort and distress with failure to respond to three months or six months lamp treatment should raise the question of operation. In such a case, Brown says, the lamp is of little value, though the lamp and the X-ray may do better. In acutely progressive intestinal disease, even if widespread, the chance of operation should be taken if the lung condition is not too active. No other treatment will overtake the disease. It is surgery or nothing.

Over against the shock of operation, there are the compensating advantages of eliminating an active focus of disease; of clearing up the diarrhoea, and of the relief of pain. Operation may be indicated even for the relief of symptoms. The surgeon asks that the intestinal disease to be suitable for operation, shall be early, localized and limited; that the small bowel shall not be too widely involved, and especially that the lung lesions shall be limited in extent and activity, and the general condition fairly good.

But we can rarely give the surgeon all he asks. We are privileged to see the earlier stages of disease in very few of our patients. Intestinal disease when we see it first is in most cases a part of a distressful, far-advanced, long-neglected general condition for which ignorance both in and out of our profession are responsible. Surgery has little to offer if it can help only an ideal early case; if it can pluck no other brands from the burning.

*Treatment Eclectic*—The choice of treatment must be eclectic. The big, obtrusive, difficult question is as to the place of surgery; the great decision, to operate or not to operate. This question a physician can not presume to finally decide. This much can be said, that operation, however successful, is but an incident in a long, patient treatment. To it all other useful forms of treatment must be added—is altogether the most promising.

Though still largely empirical treatment, its results apparently are such that it should be pushed perseveringly in all definite or even suspected cases of intestinal tuberculosis, and should not be denied, where it can be given, to even the far advanced and apparently hopeless.

*Summary*—We may conclude, then, that intestinal ulceration, tuberculosis in origin, is found in a fairly wide percentage of cases of pulmonary tuberculosis. It is likely implanted early, perhaps earlier than the lung lesion. It is almost universally present in the late stages of pulmonary disease and hastens, or perhaps causes, death.

It is treatable and even curable in its early stages. Early diagnosis, especially of disease in the small bowel, is not yet satisfactory, but much can be done by careful study of symptoms and the opaque meal and enema. Surgical treatment is suitable in some cases, but is of limited applicability. The most promising treatment at present would seem to be the ultra violet ray given by the Quartz lamp, with perhaps the X-rays as well.

Given in The Canadian Medical Association Journal, January, 1923, pages 20, 21, 22 and 23, No. 37.

